# Systematic Evaluation of Extracellular Coating Matrix on the Differentiation of Human-Induced Pluripotent Stem Cells to Cortical Neurons

**DOI:** 10.3390/ijms26010230

**Published:** 2024-12-30

**Authors:** Siyao Li, Yan Liu, Xianyang Luo, Wei Hong

**Affiliations:** Shenzhen Key Laboratory of Neuroimmunomodulation for Neurological Diseases, Shenzhen-Hong Kong Institute of Brain Science, Shenzhen Institutes of Advanced Technology, Chinese Academy of Sciences, Shenzhen 518055, China; sy.li1@siat.ac.cn (S.L.); y.liu@siat.ac.cn (Y.L.); xy.luo1@siat.ac.cn (X.L.)

**Keywords:** induced pluripotent stem cells (iPSCs), neuronal differentiation, live-cell imaging, neurite outgrowth, morphology, synaptic markers

## Abstract

Induced pluripotent stem cell (iPSC)-derived neurons (iNs) have been widely used as models of neurodevelopment and neurodegenerative diseases. Coating cell culture vessels with extracellular matrixes (ECMs) gives structural support and facilitates cell communication and differentiation, ultimately enhances neuronal functions. However, the relevance of different ECMs to the natural environment and their impact on neuronal differentiation have not been fully characterized. In this study, we report the use of four commonly used extracellular matrixes, poly-D-lysine (PDL), poly-L-ornithine (PLO), Laminin and Matrigel, which we applied to compare the single-coating and double-coating conditions on iNs differentiation and maturation. Using the IncuCyte live-cell imaging system, we found that iNs cultured on single Matrigel- and Laminin-coated vessels have significantly higher density of neurite outgrowth and branch points than PLO or PDL but produce abnormal highly straight neurite outgrowth and larger cell body clumps. All the four double-coating conditions significantly reduced the clumping of neurons, in which the combination of PDL+Matrigel also enhanced neuronal purity. Double coating with PDL+Matrigel also tended to improve dendritic and axonal development and the distribution of pre and postsynaptic markers. These results demonstrate that the extracellular matrix contributes to the differentiation of cultured neurons and that double coating with PDL+Matrigel gives the best outcomes. Our study indicates that neuronal differentiation and maturation can be manipulated, to a certain extent, by adjusting the ECM recipe, and provides important technical guidance for the use of the ECM in neurological studies.

## 1. Introduction

Induced pluripotent stem cell (iPSC)-derived neurons (iNs) have been widely used in the modeling of human developmental processes and diseases, such as Alzheimer’s disease, Parkinson’s disease, Huntington’s disease and motor neuron diseases [1,2,3,4]. The efficiency of neuronal differentiation and homogeneity of harvested neurons largely rely on induction strategies and protocols. Induced expression of transcription factors, such as Neurogenin-2 (NGN2) and ASL1, can rapidly generate a vast range of functional neurons from progenitors, stem cells, glial cells and fibroblasts with high reproducibility [5,6,7]. NGN2 overexpression alone, in combination with other transcription factors, or in the presence of small molecules has been used to generate different populations of neurons [8,9]. Despite continuous modifications, the methods for neuronal differentiation and long-term culture remain unstandardized. The timescale that is required for neuronal conversion, the purity and the morphology of the resulting neurons vary between groups and between publications from the same groups.

Nonetheless, certain common observations have emerged and it is likely that the extracellular matrix (ECM) that is applied and coated on culture vessels has a significant effect on neuronal development [10,11,12]. The ECM is a dynamic three-dimensional network of macromolecules that provides structural support for cells and tissues, maintains their physiological functions and facilitates cellular communications [13,14,15]. At the tissue level, the components of the ECM are mostly secreted by fibroblasts and are categorized into proteoglycans and fibrous proteins, including Laminin, collagen, gelatin and fibronectin [16,17]. Several types of ECM are commercially available and widely used in neuronal culture, while the selection and application of ECM in neuronal culture depends on their components, which create specific environments for desirable biological functions and cellular process [11]. For instance, cultured hippocampal neurons tend to form axons preferentially on the substrates coated with Laminin than on poly-L-lysine, suggesting that ECM or cell surface components may serve as extrinsic cues for cellular polarization [18,19,20]. This process is closely related to the activation of intrinsic integrin signaling that promotes directional microtubule assembly and axon development [18,21]. Clumping of cell bodies is a common observation in human iNs, especially in long-term culturing scenes [22,23]. Such morphological abnormality extensively affects subsequent functional assessments of single neurons, such as calcium imaging or patch clamp analysis. A recent study briefly reported that a combination of two ECM substrates could reduce the clumping of neurons, while no detailed comparisons of single coatings and double coatings were described [23]. Meanwhile, it is still not clear which among all the reported ECM substrates and their combinations is optimal for the differentiation and maturation of human iNs.

In this study, we investigated the effects of several currently used ECM coating strategies on the differentiation and maturation of human iNs. Four ECM substrates were employed (single coatings) and their combinations (double coatings) were tested. Taking advantage of the IncuCyte live-cell imaging paradigm, we found that all the four ECM ensured neuronal differentiation from iPSC when applied alone, but there were readily quantifiable differences between them. When cultured in PDL- or PLO-coated vessels, neurite outgrowth and branch points of iNs were significantly lower than with Matrigel or Laminin, while the latter two produced more straight neurites and larger cell body clumps. Four double-coating conditions produced comparable levels of neurite outgrowth and branch points with Matrigel and Laminin, but the PDL+Matrigel condition significantly reduced the clumping of neurons and enhanced neuronal homogeneity. This coating method also tended to improve dendritic and axonal development and the distribution of pre- and postsynaptic markers. These results demonstrate that extracellular matrixes contribute to the differentiation of cultured neurons and that double coating with PDL+Matrigel gives the best outcomes. Our study highlights the importance of choosing appropriate ECM for neuronal culture and that certain ECM combinations are highly recommended in some scenes.

## 2. Results

### 2.1. Single ECM Coating Has Different Effects on the Efficiency of Neuronal Differentiation

Neurons typically consist of two morphological structures: the rounded neuronal cell body (referred to as soma) and long branching processes (axons and dendrites which referred to as neurites) [24]. Neurite length and branch points are commonly used morphological indicators to evaluate neuronal differentiation and development [25]. Neurites are the protrusions extending from the neuronal soma, which can be either axons or dendrites. Neurite length refers to the distance from the neuronal soma to the end of the protrusions and is an important indicator of neuronal differentiation and growth. During neuronal differentiation, the length of these protrusions tends to increase over time. Branch points refer to the position where neurites branch or the intersection of two neurites. A single neuron has multiple protrusions, and each of these may further branch, thereby increasing the connections between neurons [26]. During neuronal maturation, the branching of dendrites becomes more and more complex, and the number of branch points could reflect synaptic connections and neuronal communications.

**Figure 1:**
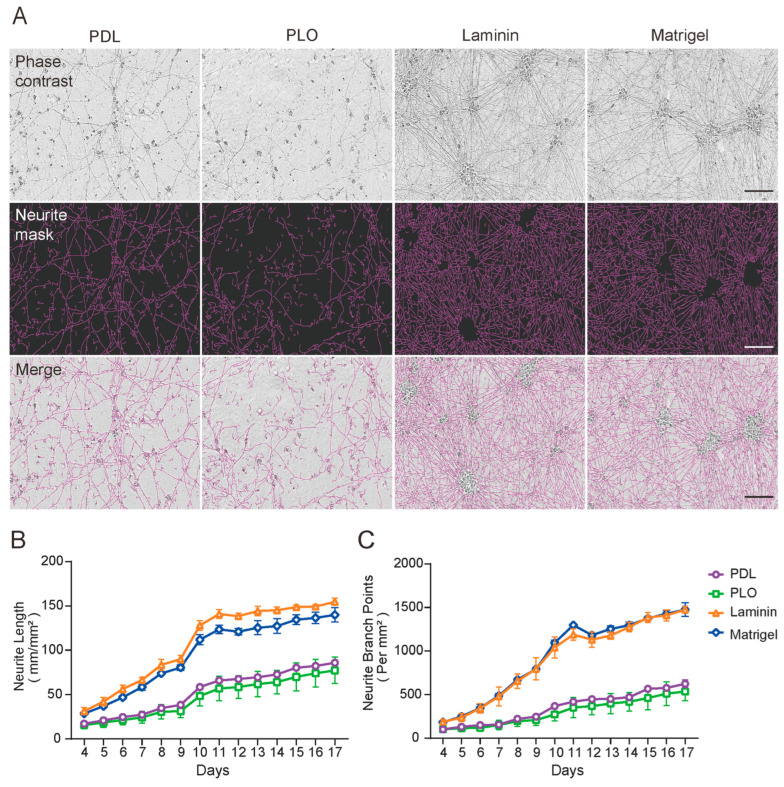
iNs cultured on Matrigel- and Laminin-coated vessels have a significantly higher density of neurite and branch points than PLO or PDL. iNs were cultured on a 96-well plate coated with a single matrix and their differentiation was monitored using the IncuCyte live-cell imaging system. (**A**) Representative images of iNs cultured for 17 days. The top panel shows phase-contrast images, the middle panel shows neurons identified by the Incucyte NeuroTrack algorithm (purple), and the bottom panel shows composite images of the identified neurons and phase-contrast field. Scale bars are 100 μm. (**B**,**C**) iNs were monitored continuously from day 4 to 17 and images were collected at 24 h intervals. The neurite length and branch points at each time points were quantified using the NeuroTrack algorithm. The values shown represent the mean (±SD) from triplicate wells for each treatment. Single coatings of Laminin (orange) and Matrigel (blue) resulted in significantly higher neurite length and branch points in iNs compared to PDL (purple) and PLO (green).

To investigate whether different ECM affect the morphology and neurite outgrowth of iNs, we firstly chose four widely used ECM for single-coating tests. We utilized the IncuCyte live-cell imaging system from Essen Bioscience as the primary measurement. At day 4 of induction, iNs were plated in ECM-coated 96-well plates and continuously monitored for 14 days. When iNs were cultured in PDL- or PLO-coated wells, we observed sparse neurite outgrowth with extensive cell debris at iN day 17 (Figure 1A). In contrast, when iNs were cultured in Laminin- or Matrigel-coated wells, we observed highly dense neurite outgrowth without visible cell debris (Figure 1A). By using NeuroTrack analysis, we were able to quantitatively measure real-time neurite outgrowth in the 2-week period. In the Laminin- or Matrigel-coated wells, neurite length and branch points increased rapidly from iN day 4 to 11 but thereafter remained nearly constant, indicating that neuronal maturation occurs (Figure 1B,C). Despite the moderate increase, the neurite length and branch points of iNs cultured in the PDL- or PLO-coated wells were far less than Laminin- or Matrigel-coated wells (Figure 1B,C). These data suggest that the differentiation and maturation of iNs, as well as their health status, may be attributed to their culture environment.

### 2.2. Double Coating with Two ECM Substrates Improves Morphological Features of iNs

Although the differentiation and maturation of iNs can be greatly improved by using Laminin- or Matrigel-coated vessels, multiple morphological abnormities of the mature neurons are non-negligible. For instance, cell somas of iNs aggregated gradually during differentiation and large clumps were extensively observed at iN day 17. In addition, when cultured in Laminin- or Matrigel-coated vessels, neurites were mostly bundle-like and straight from one neuronal cluster to another (Figure 1A). A recent study briefly descried that double-coating conditions could reduce cell toxicity, prevent clumping, and achieve an evenly distributed MAP2^+^/TUBB3^+^ neurite network [23]. Referring to this strategy, we tested four double-coating conditions, in which PLO or PDL was coated at the bottom and Laminin or Matrigel was coated on the top. iNs were plated in the coated 96-well plates and analyzed as described above.

**Figure 2:**
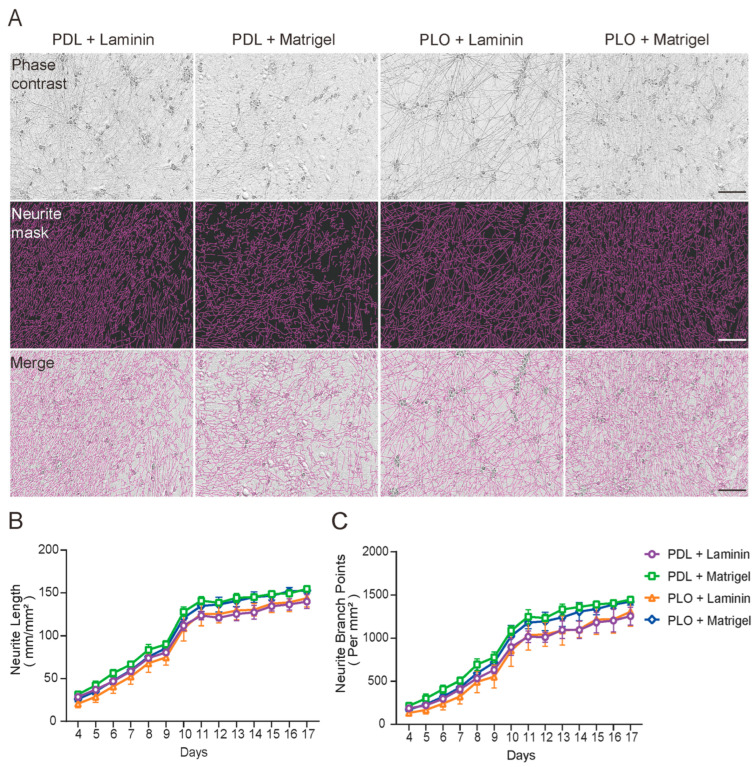
iNs cultured under different double-coating conditions yield similar and robust neurite outgrowth. iNs were cultured on a 96-well plate with double-coating matrixes and their differentiation monitored using the IncuCyte live-cell imaging system. (**A**) Representative images of iNs cultured for 17 days. The top panel shows phase-contrast images, the middle panel shows neurons identified by the Incucyte NeuroTrack algorithm (purple), and the bottom panel shows composite images of the identified neurons and the phase-contrast field. Scale bars are 100 μm. (**B**,**C**) iNs were monitored continuously from day 4 to 17 and images were collected at 24 h intervals. Neurite length and branch points at each time points were quantified using the NeuroTrack algorithm. The values shown represent the mean (±SD) from triplicate wells for each treatment. No significant differences in iNs differentiation were observed between double coatings of PDL+Laminin (purple), PDL+Matrigel (green), PLO+Laminin (orange) and PLO+Matrigel (blue).

At iN day 17, all four double-coating conditions yielded highly dense neurite outgrowth and were indistinguishable from each other (Figure 2A). NeuroTrack analysis and time-course recordings showed that neurite length and branch points increased rapidly from iN day 4 to 11 and remained nearly constant thereafter (Figure 2B,C). This common pattern was observed in all four double-coating conditions and comparable to single coating with Laminin or Matrigel (Figure 1 and Figure 2). In addition, cell debris was not seen in the iNs cultured under any of the four double-coating conditions, indicating the necessity of Laminin or Matrigel (Figure 2A).

**Figure 3:**
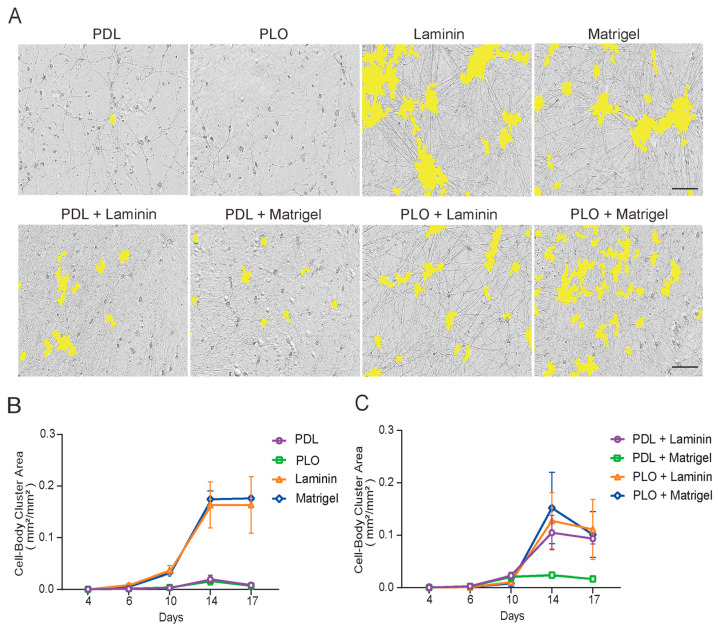
PDL+Matrigel double coating significantly reduces clumping of induced neurons. Images were collected at 2–4 days intervals from iNs day 4 to 17 and the area of cell body clusters was identified and quantified by the Incucyte NeuroTrack algorithm. (**A**) Representative images of iNs on day 17 and cell body clusters are masked (yellow). Scale bars are 100 μm. (**B**) Single-coating comparison shows that Laminin (orange) and Matrigel (blue) led to significantly more cell body clumps than PDL (purple) and PLO (green). (**C**) Among the four double-coating conditions, PDL+Matrigel (green) resulted in the lowest area of cell body clusters when compared to PDL+Laminin (purple), PLO+Laminin (orange) and PLO+Matrigel (blue). Only the cell body clusters with a size >400 μm^2^ were counted. The values represent the mean (±SD) from triplicate wells for each treatment.

Interestingly, we observed a reduction in large cell body clumps in iNs cultured under all four double-coating conditions when compared to single coating conditions with Laminin or Matrigel (Figure 3A). Using the IncuCyte 2021A software, cell body clusters with different sizes were identified unbiasedly and the ones with a size >　400 μm^2^ were counted to define neuronal clumping. iNs cultured in Laminin- and Matrigel-coated plates rapidly formed large clumps (nearly 20% area) from iN day 4 to 14 and remained constant to iN day 17. PDL- and PLO-coated plates did not yield such cell clumps (less than 3%), likely due to their unhealthy status and the occurrence of cell death (Figure 3). Although this increased with time, the area of large cell clumps in three double-coating plates (PDL+Laminin, PLO+Laminin, PLO+Matrigel) ranged from 10% to 15% at iN day 14 and 17 (Figure 3C). Most strikingly, only less than 3% of the area of PDL+Matrigel-coated wells was covered by cell body cluster masks (Figure 3C). Under this coating condition, the reduction in cell body clumping was also accompanied with a significant reduction in straight neurite bundles, indicating a possible structural rearrangement of neuronal networks.

### 2.3. Comprehensive Live-Cell Comparison of Single-Coating and Double-Coating Conditions Shows That PDL+Matrigel Is the Optimal Substrate for Long-Term Neuronal Culture

**Figure 4:**
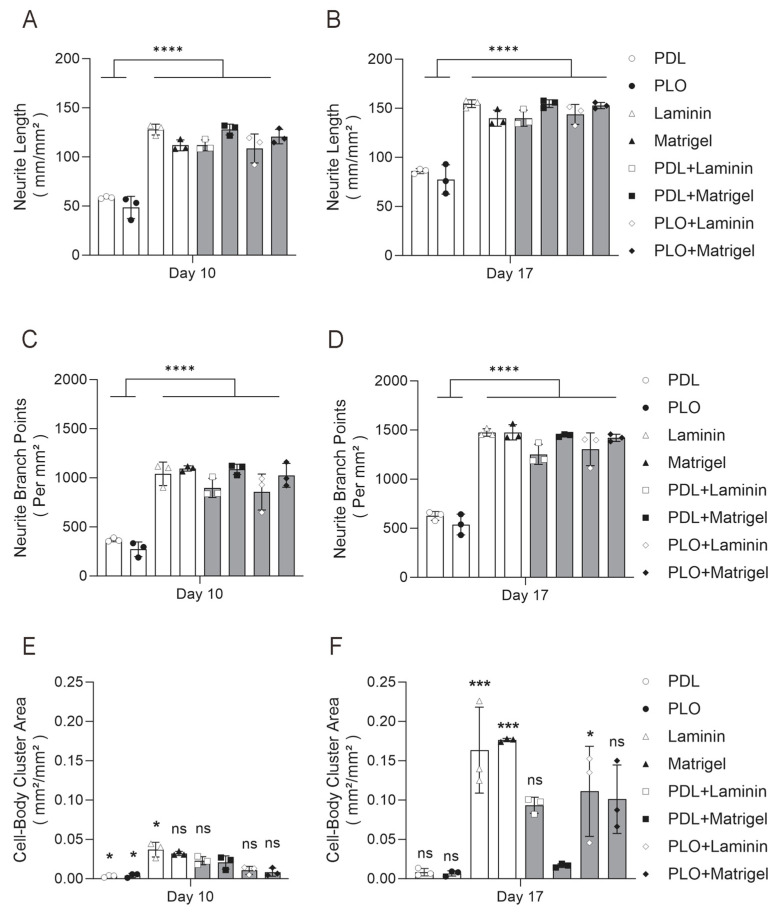
Comprehensive live-cell comparison of single-coating and double-coating conditions shows that PDL+Matrigel is the optimal substrate for long-term neuronal culture. Images collected at iNs day 10 and 17 (in Figure 1, Figure 2 and Figure 3) were statistically compared for their neurite length, neurite branch points and cell body cluster area. (**A**,**B**) Neurite length of iNs cultured at day 10 and 17 under four single-coating and four double-coating conditions. (**C**,**D**) Neurite branch points of iNs cultured at day 10 and 17 under four single-coating and four double-coating conditions. (**E**,**F**) Cell body cluster area of iNs cultured at day 10 and 17 under four single-coating and four double-coating conditions. Data represent the mean values (±SD) from triplicate wells for each condition. Single-coating conditions included Laminin, Matrigel, PDL and PLO. Double-coating conditions included PDL+Matrigel, PDL+Laminin, PLO+Laminin and PLO+Matrigel. Statistical analysis was conducted using one-way ANOVA followed by Tukey’s multiple comparison test. Significant differences are denoted as **** *p* < 0.0001; *** *p* < 0.001; * *p* < 0.05; ns (not significant) *p* > 0.05.

To provide a better overview of neuronal differentiation and morphological features, we statistically compared the neurite length, branch points and cell body cluster area of iNs cultured under the four single-coating and four double-coating ECM conditions. Two key time points, iN day 10 and 17, represented the middle-term and long-term differentiation stages. At iN day 10, PDL and PLO single-coating conditions (each ~50 mm/mm^2^) produced a significantly lower density of neurite length than the Laminin and Matrigel single-coating and all four double-coating conditions (all ranged from 110 to 130 mm/mm^2^) (Figure 4A). No significant difference was detected between the latter six conditions. The same degree of differences remained detectable until iN day 17, although the neurite length in all groups increased by ~50% (Figure 4B). At iN day 10, the number of neurite branch points of iNs cultured under PDL and PLO single-coating conditions only reached 250–400 per mm^2^, while the other six conditions ranged from 800 to 1100 per mm^2^ (Figure 4C). Again the same degree of differences remained detectable until iN day 17 and no significant difference was observed between the latter six groups (Figure 4D).

Excessive aggregation of cell bodies may impair neuronal differentiation and development by restricting neurite outgrowth or preventing synaptic connections. Based on the iN images and quantitative data presented in Figure 1, Figure 2 and Figure 3, we here focused on the optimal coating condition, for which a combination of PDL and Matrigel was used. Thus, statistical analysis of the cell body cluster area was mainly performed between the PDL+Matrigel double-coating group and the seven other groups. At iN day 10, approximately a 2.5% area was covered by large cell body clusters (>400 μm^2^) in the PDL+Matrigel double-coating group, which was significantly higher than PDL and PLO alone and lower than Laminin (Figure 4E). At iN day 17, the PDL+Matrigel double-coating group demonstrated a 2.5% area of large cell body clusters, which was not significantly different to PDL and PLO. As mentioned before, this phenomenon may be attributed to the impairment of cell health and the occurrence of cell death induced by PDL and PLO in long-term neuronal culture. Nonetheless, all of the other coating conditions, Laminin, Matrigel, PDL+Laminin, PLO+Laminin and PLO+Matrigel, produced severe neuronal clumps at iN day 17 (Figure 4F).

Taken together, these live-cell imaging data suggest that PDL+Matrigel double coating is the optimal substrate condition for long-term neuronal culture. Beneficial responses can be quantitatively proved though several readouts, such as more efficient neurite growth, improved branching complexity, and significantly less cell body aggregation and clumping, which are virtually the key drivers of neuronal maturation and network formation.

### 2.4. Double Coating with PDL+Matrigel Enhances Neuronal Purity and Tends to Improve the Expression of Neuronal and Synaptic Markers

Live-cell imaging analysis provides macroscopical and high-throughput measurements of several parameters during neuronal differentiation. While the efficiency of neuronal conversion and expression of neuronal markers are not clear, considering the occurrence of cell debris and neuronal clumping, as well as the diverse pattern of neurite outgrowth among eight ECM coating conditions, we believe that particular molecular and cellular signatures are tightly linked to them. As such, we characterized the expression of several neuronal markers by immunostaining.

**Figure 5:**
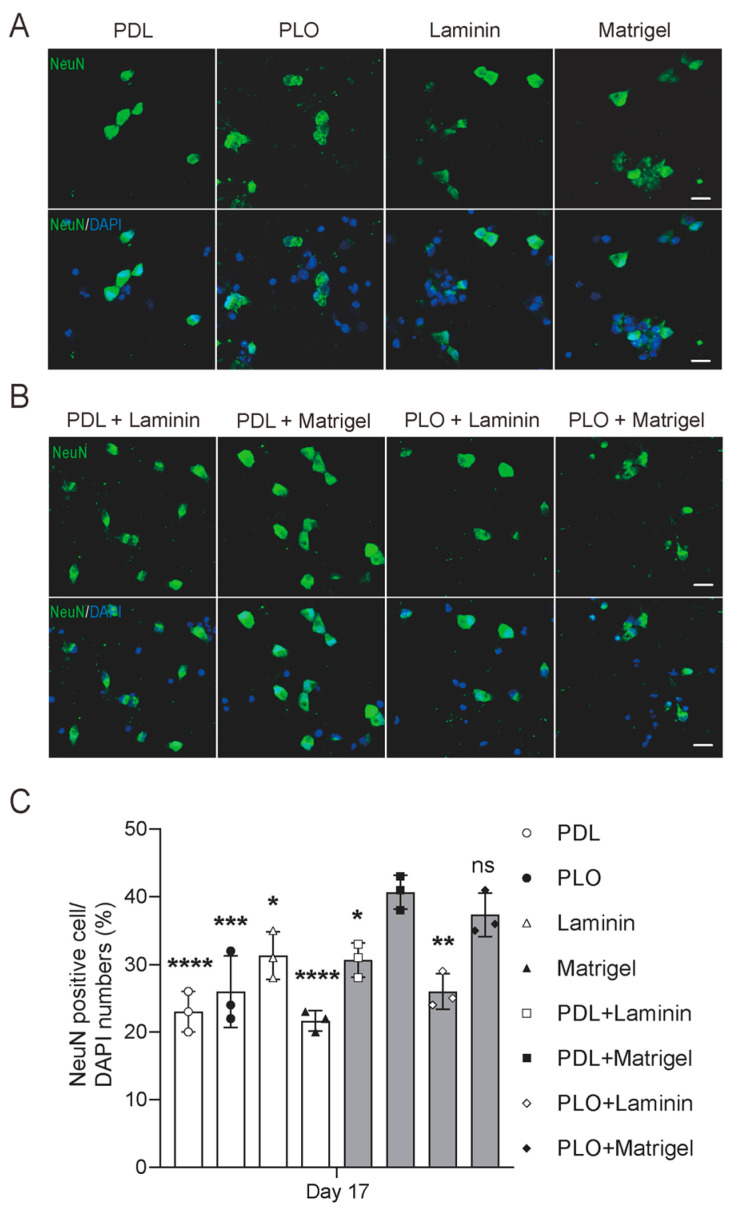
PDL+Matrigel double-coating condition enhances the purity of induced neurons. At 17 days post-induction, iNs were fixed and stained for the mature neuronal marker NeuN (green). Cell nuclei were stained with DAPI (blue). Scale bar: 20 μm. (**A**,**B**) Representative immunofluorescence images collected at iNs day 17 show co-localization of NeuN and DAPI. (**C**) Quantification of NeuN-positive cells versus total cells shows the purity of iNs cultured under different coating conditions. Data represent the mean values (±SD) from triplicate wells for each condition. Single-coating conditions included PDL, PLO, Laminin and Matrigel. Double-coating conditions included PDL+Matrigel, PDL+Laminin, PLO+Laminin and PLO+Matrigel. Statistical analysis was conducted using one-way ANOVA followed by Tukey’s multiple comparison test. Significant differences are denoted as **** *p* < 0.0001, *** *p* < 0.001, ** *p* < 0.01, * *p* < 0.05, ns (not significant) *p* > 0.05.

Neuronal nuclei (NeuN) is a well-recognized marker that is detected exclusively in post-mitotic neurons and indicates neuronal maturity. To confirm the neuronal conversion efficiency under different ECM coating conditions, we employed immunocytochemistry to label mature neurons with NeuN at iN day 17 and measured the mRNA levels of NeuN (Figure 5A,B). Our results showed that although there is no significant difference in mRNA levels, the percentage of NeuN-positive cells is different (Figure S1A). The PDL+Matrigel double-coating condition yielded a significantly higher induction efficiency of iPSCs into iNs compared to other coating conditions (Figure 5C). The percentage of NeuN-positive cell numbers versus total DAPI-stained nuclei numbers was significantly higher in the PDL+Matrigel and PLO+Matrigel coating groups than others, despite the fact that the latter two had no significant difference (Figure 5C). These data confirm the discovery that the PDL+Matrigel double-coating condition is the optimal substrate condition for converting iPSCs to iNs.

**Figure 6:**
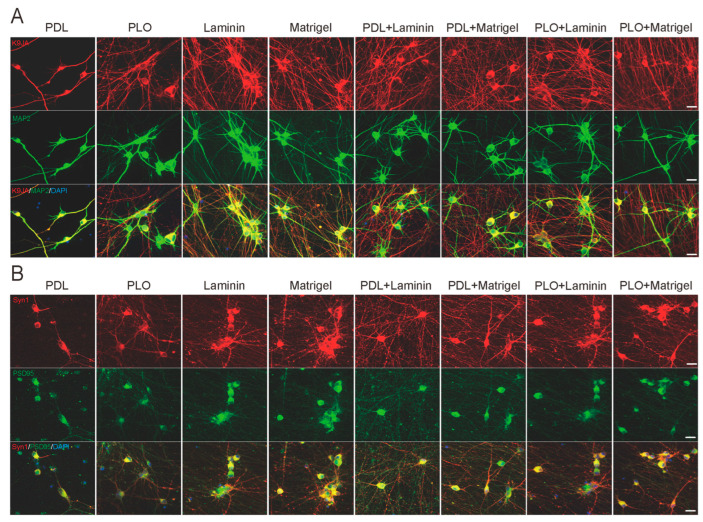
Expression of neuronal and synaptic markers tends to be improved in iNs cultured under PDL+Matrigel double-coating condition. At 17 days post-induction, iNs were fixed and stained for an array of neuronal markers. (**A**) Representative images showing immunostaining for axons (K9JA, red) and dendrites (MAP2, green) under eight coating conditions. (**B**) Representative images showing immunostaining for presynaptic marker Synapsin-1 (Syn1, red) and postsynaptic marker Postsynaptic Density Protein 95 (PSD-95, green) under eight coating conditions. Scale bar: 20 μm.

To further confirm the differentiation and maturation of iNs at the molecular level, we performed immunocytochemistry assessments of several neuronal and synaptic markers such as microtubule-associated protein MAP2 (dendritic marker) and Tau (axonal marker), as well as Synapsin-1 (Syn1, presynaptic marker) and Postsynaptic Density Protein 95 (PSD95, postsynaptic marker). Similarly to the above live-cell imaging results, all coating conditions except PDL and PLO produced highly dense Tau-positive axons (K9JA) and MAP2-positive dendrites. It is worth mentioning that more equally distributed axons and dendrites were observed in the groups with less neuronal clumps, especially in the PDL+Matrigel group (Figure 6A). At the synaptic level, we also did not see a significant difference in Syn1 and PSD95 mRNA levels between the eight coating conditions (Figure S1B,C). However, the immunostaining results showed that both Syn1- and PSD95-positive puncta were improved and distributed more equally in iNs cultured under double-coating conditions. Specifically, the cellular distributions of Syn1 and PSD95 in the PDL+Matrigel group were much more equal than others, indicating the possibility of functional transportation of the key components from soma to synapse (Figure 6B). These data suggest that ECM may improve the distribution of neuronal proteins instead of increasing their expression level. These data also verify the live-cell imaging assessments that neuronal differentiation and synaptic maturation can be remarkably improved by optimal ECM coating conditions, which refers to PDL+Matrigel in our case.

To determine whether different coating conditions affect the iPSC status that substantially modulates the ultimate neuronal differentiation, we also validated the effects of different ECM coating conditions on iPSC self-renew and pluripotency maintenance. Our data showed that iPSC cultured on a Matrigel-coated vessel showed regular colony morphology and reached 25% confluence at day 5, while iPSC in PDL-, PLO- and Laminin-coated vessels did not grow into such clusters and only had less than 10% confluence (Figure S2A,C). All the four double-coating conditions produced more and larger iPSC colonies than single-coating conditions (30–50% confluence at day 5), among which the PDL+Matrigel group was the highest and had 90% confluence (Figure S2B,D). With regard to neuronal markers and stem cell markers, we found that the mRNA levels of NeuN, Syn1, PSD95, SOX2 and OCT4 in iPSC are highly diverse in the iPSC cultured under different coating conditions, and that PDL+Matrigel was the most comparable to widely used Matrigel (Figures S1D–F and S3). In fact, in the present study, we did not directly plate iPSC into the vessels coated with eight different coating matrixes. Instead, day 4 iNs were plated into a 96-well plate and no iPSC status was involved in our study since the cells were already pre-mature neurons at the time they were plated. These data suggest that coating matrixes do have significant effects on the maintenance of iPSC pluripotency, but the PDL+Matrigel double-coating strategy does not cause a noxious effect.

## 3. Discussion

iPSCs provide a powerful tool to study human development processes, model disease progression and facilitate drug discovery. Disease-specific human iPSCs can be differentiated into relevant cell types that potentially recapitulate disease pathology on the basis of human background, which are more accurate than existing animal models in some respects and expected to overcome difficulties associated with species differences [27,28]. In the field of the central nervous system, iNs such as dopaminergic neurons, glutamatergic neurons and cholinergic neurons have been widely used in the study of neuronal disorders and neurodevelopment [1,2,29]. Certainly, accurate dissection of the cell biology in disease modeling relies on a developmentally mature cell phenotype [30]. In recent years, researchers have made substantial progress in the development of efficient differentiation methods for iNs, which mostly involve extrinsic growth factors but require complex and time-consuming culture procedures [31]. Here, we show that neuronal differentiation and maturation can be improved by simply adjusting the ECM substrate in neuronal culture.

The ECM not only provides structural support but also contains multiple cytokines and proteins that are critical for reconstructing the physiological structure and function of tissues and cells [32]. To systematically validate the utility of ECM in neuronal culture, we employed a high throughput live-cell imaging paradigm to assess the differentiation efficiency and morphology of human iNs [33]. Four ECM substrates were investigated in the present study, Laminin, Matrigel, PDL and PLO. Laminin is a multi-domain trimeric glycoprotein and the main non-collagenous component of basal lamina; it promotes cell adhesion, migration and differentiation by binding to integrin receptors on the cell surface [34,35] and triggers axon formation by promoting microtubule assembly and increasing its stabilization [18]. Matrigel is a solubilized basement material from mouse sarcoma and is rich in extracellular proteins such as Laminin, collagen IV, heparan sulfate proteoglycans and growth factors [36]. Matrigel not only promotes cell adhesion but also regulates cell behavior through multiple signaling pathways; thus, it is the most widely used substrate to support the growth and differentiation of stem cells and neurons [37,38]. The coating of synthetic polymers (poly amino acids) facilitates the attachment of both cells and proteins [14]. Poly-amino acids like PDL and PLO create a positive charge on polystyrene and increase the positively charged sites available for cell binding [39]. Taking advantage of the unbiased IncuCyte live-cell imaging analysis, we were able to obtain quantitative insights into the effects of different ECM coating strategies on the differentiation and maturation of human iNs. Specifically, our assessments focus on three critical parameters: neurite length, neurite branch points and cell body cluster area. Neurite length is a measurement of neurite outgrowth, reflecting the growth and maturation of neurons. Branch points represent the complexity of neurite branching and are crucial for the formation of complex neuronal networks.

When neurons were cultured in Laminin- or Matrigel-coated vessels, we observed a significantly higher density of neurite length and branch complexity compared to PDL or PLO (Figure 1 and Figure 4). This phenomenon is consistent with prior studies that demonstrated the positive impact of Laminin and Matrigel on neuronal development and facilitation of growth cone dynamics and synaptogenesis in the presence of key ECM components [40]. These data indicate that Laminin and Matrigel are more supportive for neuronal differentiation and maturation, likely due to their ability to better mimic the native neural extracellular matrix and provide a more conducive environment for neuronal outgrowth. However, in Laminin- or Matrigel-coated vessels, neurites were mostly bundle-like and straight from one neuronal cluster to another (Figure 1, Figure 3 and Figure 4). Overall, our data showed that single coating with Laminin or Matrigel is sufficient for neuronal differentiation and long term culture but generates abnormal morphology of iNs. Meanwhile, single coating with PDL or PLO does not cause cell clumping but is also not supportive for neuronal differentiation and survival. Thus, we conclude that none of the four single-coating conditions is able to provide satisfactory readouts of neuronal differentiation and maturation.

Corresponding to a recent study which utilized double-coating conditions for neuronal culture [23], we integrated the readouts from the above four single-coating conditions and tested their combinations. Four double-coating conditions were tested, each with PLO or PDL coated at the bottom and Laminin or Matrigel coated on the top. By live-cell imaging analysis, we found that all four double-coating conditions produce similar levels of neurite outgrowth and branch points compared to Matrigel and Laminin (Figure 1, Figure 2 and Figure 4). This phenomenon suggests that once sufficient biological signals from the ECM are achieved, the additional signals do not translate into functional gains. Therefore, an extra coating of Matrigel or Laminin on top of PDL or PLO does not produce more neurite outgrowth.

Immunostaining data showed that NeuN-positive cells are more abundant in the iNs cultured under all the four double-coating conditions than single-coating conditions, among which PDL+Matrigel is the most prevailing condition (Figure 5). Remarkably, double-coating conditions also produced less cell body aggregation and clumping than single coating (Figure 3), which is associated with more evenly distributed synaptic markers (Figure 6). It is necessary to declare that double-coating strategies may only improve the distribution of neuronal proteins instead of increasing their expression level. In fact, the morphological abnormities of differentiated neurons are non-negligible. Neurons require sufficient space for neurite outgrowth during differentiation and development, which is the structural basis for neuronal communication. Excessive cell body clumping may restrict neurite extension and connection, prevent transportation of neuronal proteins and eventually disrupt the formation of the neuronal network and induce neurodegeneration or even cell death. The superior performance of the PDL+Matrigel combination highlights the importance of ECM composition in spatial cell organization. This phenomenon also reflects a possible but unique interaction between the structural support of PDL and the biochemical cues from Matrigel, resulting in a more favorable environment for neuronal differentiation [16,41]. This is a crucial observation, as it underscores the importance of both structural and biochemical factors in optimizing the conversion of iPSCs to neurons. Given the complexity of neuronal differentiation, a more diverse ECM environment, such as PDL+Matrigel, may strike an optimal balance between promoting cell survival, reducing aggregation and enhancing neurite outgrowth. In addition, human iNs generally display limited synaptic maturation, with considerably slower development than that of rat primary neurons. Our data provide a desirable approach to facilitate synaptic maturation of iNs by simply manipulating the ECM recipe. However, it is possible that the same coating condition may have different impacts on the differentiation of different types of neurons or the same type of neurons induced by different protocols. Thus, we need to declare that a particular recipe may not be consistently perfect and strongly recommend examining the optimal coating condition for each type of neuron prior to experiments.

Except for neuronal differentiation, ECM manipulation may also applicable to cell morphological maintenance or cell fate determination. For instance, our data showed that PDL, PLO and Laminin are not suitable for iPSC culture but double-coating conditions can accelerate iPSC proliferation, among which PDL+Matrigel has the strongest effect. In addition, the PDL+Matrigel coating condition did not have a noxious effect on the maintenance of iPSC pluripotency, as determined by several stem cell markers. Thus, we conclude that such double-coating strategy may be potentially applicable in different cell culture systems.

## 4. Materials and Methods

### 4.1. Reagents and Chemicals

A 0.1 mg/mL Poly-D-lysine (PDL) solution was purchased from Gibco, a 0.01% Poly-L-ornithine (PLO) solution was purchased from Sigma-Aldrich, Laminin was purchased from Thermo Fisher and Matrigel was purchased from Corning. All the four reagents were thawed slowly on ice, aliquoted into adequate volumes under sterile conditions and stored at −20 °C until use. All other chemicals and reagents were of the highest purity available and unless indicated otherwise were obtained from Sigma-Aldrich (St. Louis, MO, USA).

### 4.2. Plasmids and Lentivirus

Plasmids pTet-O-Ngn2-puro (Addgene plasmid #52047) and Tet-O-FUW-EGFP (Addgene plasmid #30130) were gifts from Marius Wernig [5,8]. Plasmid FUdeltaGW-rtTA (Addgene plasmid #1978) was a gift from Konrad Hochedlinger [6]. All the lentiviruses used in this study were packaged and titers were determined by Taitool (Shanghai Taitool Bioscience Co., Ltd., Shanghai, China).

### 4.3. Production of Induced Neurons (iNs) from Human iPSCs

Human iPSC line DYR0100 (derived from the foreskin fibroblast cell line SCRC-1041) was kindly provided by Stem Cell Bank, Chinese Academy of Sciences. iPSCs were maintained in mTeSR media on Matrigel and passed every 4–5 days at a split ratio from 1:6 to 1:8 using Accutase (STEMCELL Technologies, Vancouver, BC, Canada). Neuronal differentiation was performed via a doxycycline-induced Neurogenin 2 (NGN2) system as previously described [8,42]. Endogenous NGN2, GFP and rtTA genes were introduced by lentivirus. For viral infection, iPSCs were plated at a density of 95,000 cells/cm^2^ into a 12-well plate and lentiviruses were added after 24 h with the following multiplicity of infection (MOI): pTet-O-NGN2-puro (MOI = 5), Tet-O-FUW-eGFP (MOI = 5) and Fudelta GW-rtTA (MOI = 3). Cell culture medium was replaced after 24 h and cells maintained in mTeSR media. To induce NGN2 expression, doxycycline was added on “iN day 1” at a concentration of 2 μg/mL. On iN day 2, puromycin was added at 10 mg/mL and was maintained in the media at all times thereafter. On iN day 4, cells were frozen down or directly plated on ECM-coated Greiner 96-well microclear plates and maintained in media consisting of Neurobasal medium (Gibco, Waltham, MA, USA), Glutamax, 20% Dextrose, MEM-NEAA and B27 and BDNF, CNTF and GDNF (PeprpTech, Rocky Hill, NJ, USA), each at a concentration of 10 ng/mL.

### 4.4. Single Coating and Double Coating of ECM

For single coating, Greiner microclear 96-well plates were coated with 0.1 mg/mL of PDL or 0.01% PLO and incubated at 37 °C overnight. Laminin and Matrigel were thawed on ice and diluted to 3.3 µg/mL and 28 µg/mL with ice-cold DMEM/F12 (Gibco, Waltham, MA, USA), added to plates and incubated at 37 °C for 2 h. Thereafter, each well was rinsed twice with 100 µL D-PBS (Servicebio Technology, Wuhan, China) and used for cell plating. For double-coating tests, plates were coated with PDL or PLO according to the above procedures and Laminin or Matrigel were coated on top. Four double-coating conditions were investigated in this study: PDL+Laminin, PDL+Matrigel, PLO+Laminin and PLO+Matrigel.

### 4.5. Characterization of iNs by IncuCyte Live-Cell Imaging System

For IncuCyte live-cell imaging and immunostaining assessments, day 4 iNs were plated at 4000/8000 cells/well on ECM-coated Greiner microclear 96-well plates. Prior studies indicated that neurite outgrowth and expression of neural markers reached near maximal levels by iN day 14 [33]. Thus, to compare the effects of different ECM coating strategies on the morphology and neurite outgrowth in the current study, iNs were monitored continuously from iN day 4 to 17 using the IncuCyte S3^®^ live-cell imaging system (Essen Bioscience, Ann Arbor, MI, USA). Phase contrast images were collected at 24 h intervals for a total of 14 days and analyzed using the NeuroTrack analysis algorithm. Neurite processes and cell bodies were automatically defined by the analysis and data were subsequently acquired for neurite length, neurite branch points and cell body cluster area. Typical settings were Segmentation Mode = Brightness; Segmentation Adjustment = 1.2; Cell body cluster filter = minimum 400 μm^2^; Neurite Filtering = Best; Neurite sensitivity = 0.4; Neurite Width = 2 μm.

### 4.6. Immunohistochemistry

At the endpoint of the experiment, iNs were fixed, stained and used for confocal microscopy. Cells were fixed in 4% paraformaldehyde (PFA) (Biosharp, Hefei, China) and 4% sucrose for 20 min at room temperature and then rinsed twice with PBS. Thereafter, cells were incubated with 0.1 M glycine for 10 min and washed twice with PBS. Cells were then permeabilized with 0.25% Triton X-100 in PBS for 5 min at room temperature and washed twice with PBS. Cells were blocked with 3% BSA in PBS for 2 h at room temperature. For immunostaining, iNs were incubated with each primary antibody (Anti-NeuN antibody, Abcam, Waltham, MA, USA; Anti-tau antibody (K9JA), DAKO, Denmark; Anti-MAP2, Abcam, USA; Anti-PSD95, Abcam, USA; Anti-Synapsin 1, Abcam, Waltham, MA, USA) at 4 °C overnight. Cells were again rinsed 3 times with PBS and incubated with fluorescence-conjugated secondary antibody (Goat Anti-Rabbit IgG H&L; Goat Anti-Mouse IgG H&L; Goat Anti-Chicken IgY H&L, Abcam, Waltham, MA, USA) for 1 h at room temperature. Finally, iNs were incubated with DAPI (1 μg/mL in PBS, Beyotime, Shanghai, China) for 15 min, rinsed 3 times with PBS, and then visualized using an Olympus confocal (SpinSR10) microscope (Olympus, Tokyo, Japan).

### 4.7. Statistical Analysis

All statistical analyses were performed using Prism10 (GraphPad Software). Differences between groups were tested with one-way ANOVA and Tukey’s test.

## 5. Conclusions

Our findings indicate that the combination of PDL+Matrigel provides multiple advantages in neuronal differentiation and maturation, including a high density of neurite outgrowth and branches, less cell body clumps, a high purity of neurons, abundant axonal and dendritic structures, and equally distributed synaptic proteins. These insights are valuable for optimizing the in vitro culture conditions of iPSC-derived neurons and could have broader implications for developing neural tissue engineering and regenerative medicine strategies. A major limitation of the current study is the lack of knowledge regarding the molecular mechanism of how the ECM modulates neuronal differentiation and maturation. One possibility is that the ECM regulates the transcription factors that are capable of driving the expression of neuronal proteins and enables the transportation of essential components from soma to synapses. In future studies, it will be important to determine the molecular mechanisms underlying these effects so as to further refine ECM combinations for specific neuronal subtypes.

## Data Availability

Data are contained within this article.

## Supplementary Materials

The following supporting information can be downloaded at: https://www.mdpi.com/article/10.3390/ijms26010230/s1.
