# Supplementary material for: Systematic Evaluation of Extracellular Coating Matrix on the Differentiation of Human-Induced Pluripotent Stem Cells to Cortical Neurons
Source: Int J Mol Sci. 2024 Dec 30;26(1):230. doi: 10.3390/ijms26010230 (PMC11720352; doi:10.3390/ijms26010230)
Supplement: Supplementary file 1 [file ijms-26-00230-s001.zip › ijms-3325893-supplementary.pdf]

Supplementary Materials and Methods

Quantitative real-time PCR

Total RNA of iPSC or induced neurons was extracted using TransZol Up Plus RNA Kit (TransGen Biotech, China) and reverse transcribed into cDNA with the Hifair®Ⅲ 1st Strand cDNA Synthesis SuperMix for qPCR (YEASEN, China). Quantitative real-time PCR was performed using Hieff UNICON® Universal Blue qPCR SYBR Green Master Mix (YEASEN, China) reagent and QuantStudio 7 Flex (Thermo Fisher). Quantification was performed using a relative standard curve and β-actin was used as an internal control. Primers used in the current study are listed as follows:

Genes	Forward primer	Reverse primer
β-actin	CACCATTGGCAATGAGCGGTTC	AGGTCTTTGCGGATGTCCACGT
SOX2	CAGCATGTCCTACTCGCAGCAG	CTGGAGTGGGAGGAAGAGGTAA
OCT4	GTGGTCCGAGTGTGGTTCTGTAAC	CCCAGCACCTCAAAATCCTCTC
NeuN	TACGCAGCCTACAGATACGCTC	TGGTTCCAATGCTGTAGGTCGC
SYN1	CGATGCCAAATATGACGTGCGTG	AGCATCGCAGAGCCAGTATTGG
PSD95	TCCACTCTGACAGTGAGACCGA	CGTCACTGTCTCGTAGCTCAGA

Characterization of iPSC by IncuCyte live-cell imaging system

For IncuCyte live-cell imaging, iPSC were plated on ECM-coated Greiner microclear 96-well plates. To compare the effects of different ECM coating strategies on cell confluence, iPSC were monitored continuously for 5 days using the IncuCyte S3® live-cell imaging system (Essen Bioscience, Ann Arbor, MI). Phase contrast images were collected at 12 hours intervals for a total of 5 days and analyzed using the continuously Basic Analyzer paradigm. Cell bodies were automatically defined by the analysis and data subsequently acquired for confluence. Cell body cluster filter = minimum 2000 μm^2.

Supplementary Figures

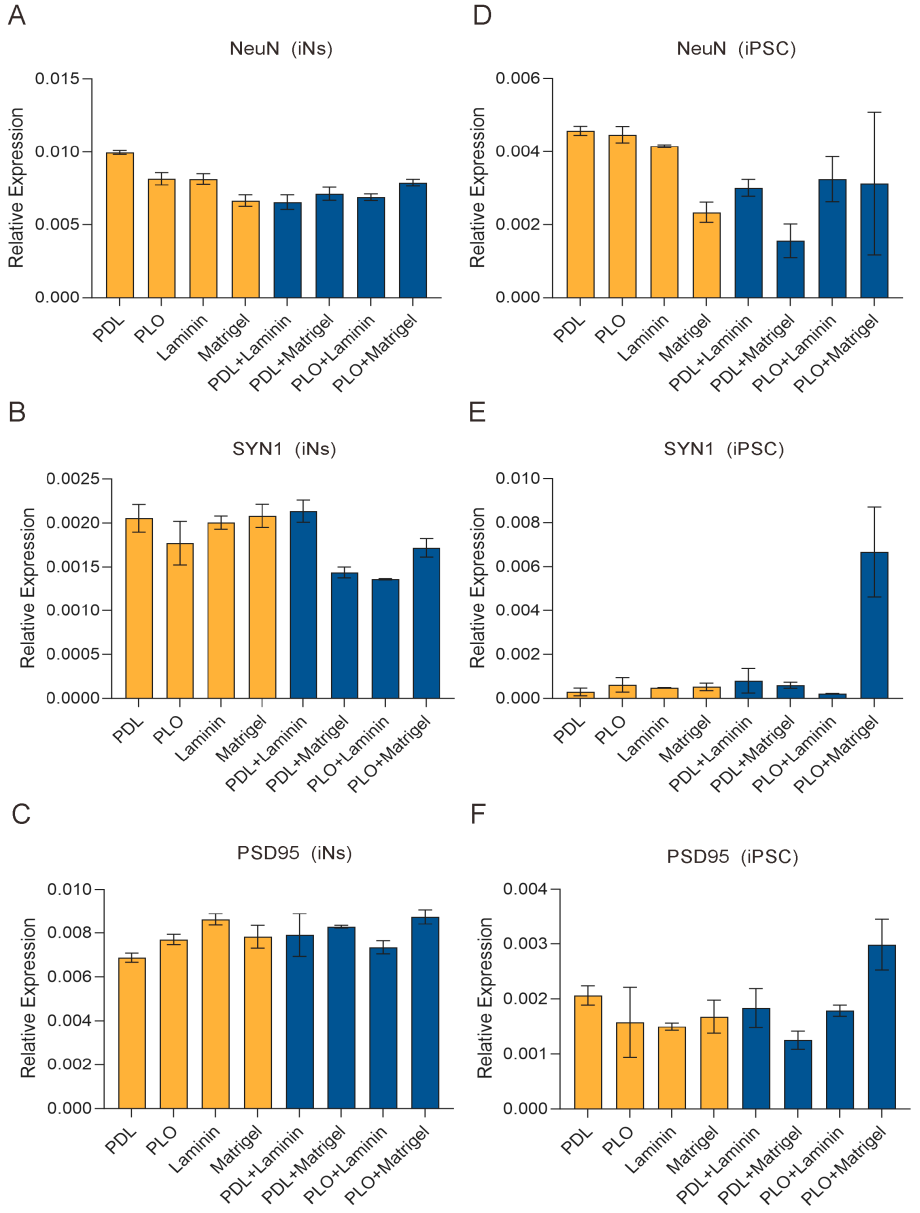

Supplementary Figure 1. Expression of neuronal proteins in iNs cultured under eight different coating conditions. Relative mRNA levels of NeuN (**A**), SYN1 (**B**) and PSD95 (**C**) normalized to β-actin in induced neurons (iNs) quantitated by qPCR. Relative mRNA levels of NeuN (**D**), SYN1 (**E**) and PSD95 (**F**) normalized to β-actin in iPSC quantitated by qPCR. Single-coating conditions and double-coating conditions are labeled in orange and dark blue respectively.

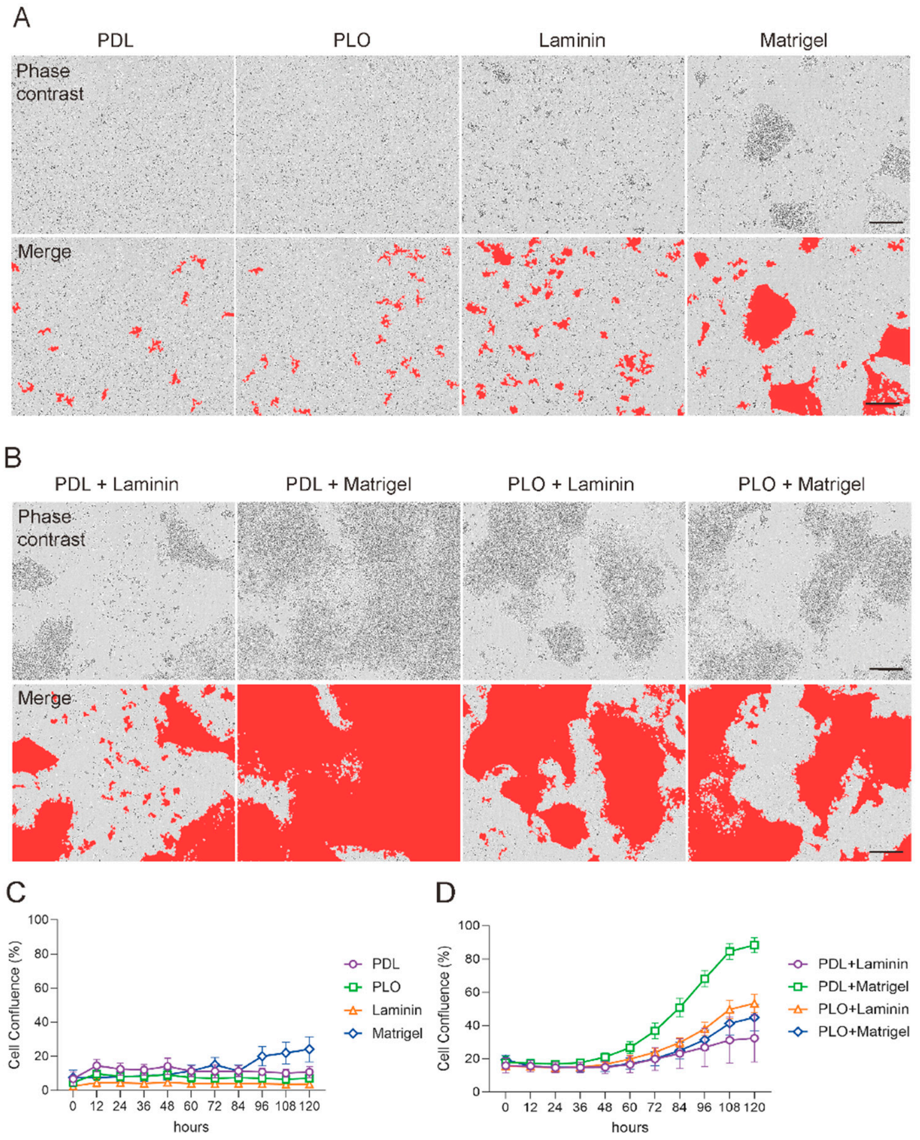

Supplementary Figure 2. Morphology and growth curve of iPSCs culture under eight coating conditions. Human iPSCs were cultured on a 96-well plate coated with single or double matrix and monitored using IncuCyte live-cell imaging system. (**A, B**) Representative images of iPSCs cultured for 5 days under single and double coating conditions. The top panel shows phase-contrast images and the lower panel shows cells identified by the Incucyte Basic Analyzer (red). Scale bars are 100 µm. (**C, D**) iPSCs were monitored continuously for 5 days and images collected at 12 hours intervals. Cell confluence at each time points were quantified using the Basic Analyzer. The values shown represent the mean (± SD) from six wells for each treatment.

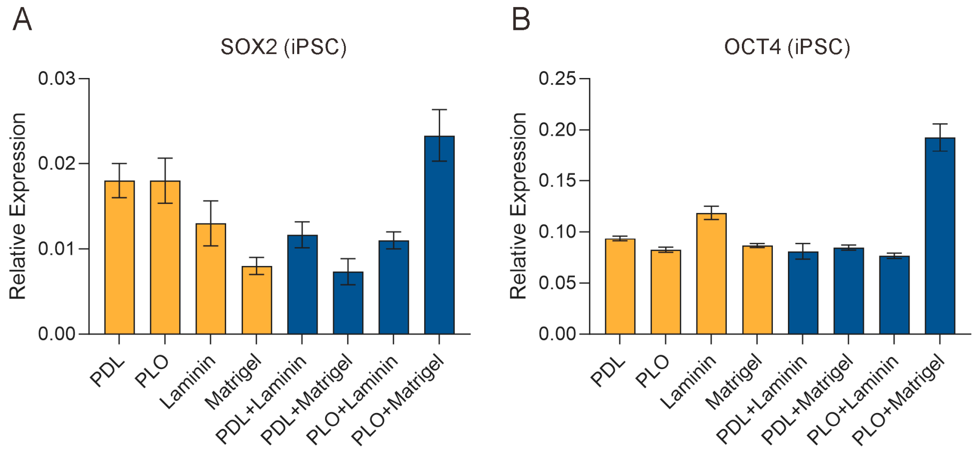

Supplementary Figure 3. Gene expression of stem cell markers in undifferentiated and differentiated neurons cultured under eight coating condition. Relative mRNA level of SOX2 (**A**) and OCT4 (**B**) normalized to β-actin in iPSCs quantitated by qPCR. Single coating conditions and double coating conditions are labeled in orange and dark blue.

References

1. Jin, M., B. O'Nuallain, W. Hong, J. Boyd, V. N. Lagomarsino, T. T. O'Malley, W. Liu, C. R. Vanderburg, M. P. Frosch, T. Young-Pearse, D. J. Selkoe and D. M. Walsh (**2018**). "An in vitro paradigm to assess potential anti-Abeta antibodies for Alzheimer's disease." *Nat Commun* 9(1): 2676.
2. Zhang, Y., C. Pak, Y. Han, H. Ahlenius, Z. Zhang, S. Chanda, S. Marro, C. Patzke, C. Acuna, J. Covy, W. Xu, N. Yang, T. Danko, L. Chen, M. Wernig and T. C. Sudhof (**2013**). "Rapid single-step induction of functional neurons from human pluripotent stem cells." *Neuron* 78(5): 785-798.
